# Enhancing a Mid-Wave Infrared Fourier Transform Hyperspectral Imager for Explosions

**DOI:** 10.3390/s26165033

**Published:** 2026-08-08

**Authors:** James T. Stofel, Kody A. Wilson, Martin Larivière-Bastien, Anthony L. Franz, Michael L. Dexter

**Affiliations:** 1 Center for Technical Intelligence Studies and Research, Air Force Institute of Technology, 2950 Hobson Way, Wright-Patterson AFB, Dayton, OH 45433, USA; kody.wilson.4@au.af.edu (K.A.W.); anthony.franz.3@au.af.edu (A.L.F.); michael.dexter.2@au.af.edu (M.L.D.); 2 Telops, Inc., 100-2600 Avenue St Jean Baptiste, Québec City, QC G2E 6J5, Canada; m.lariviere-bastien@exosens.com

**Keywords:** hyperspectral imager, Fourier-transform spectroscopy, Fourier-transform infrared, scene change artifacts, remote sensing, explosions

## Abstract

Capturing reliable hyperspectral imager data at a meaningful frame rate for explosions and other fast-changing scenes is not possible in the mid-wave infrared region under traditional sensor operating configurations and processing techniques, which typically have frame rates on the order of 0.5–2.0 Hz. To combat these shortcomings, the scene acquisition parameters were tailored for explosions and a new method for processing optical signatures of fast transient scenes with Fourier-transform infrared hyperspectral imagers was developed. For this technique, the instrument was first configured to collect asymmetric interferograms while optimizing the number of measurement points on the short side of the interferogram. Additionally, pixel-wise zero path distance offset and phase corrections were applied to the interferograms, a reduced spectral resolution of 8 cm^−1^ was selected, and the window size was narrowed to 32 × 64 pixels while using a lens with a wide field of view. The smooth offset correction for scene change artifacts was then applied in post-processing to address any remaining artifacts in the Fourier-transformed spectra. These procedures yielded a 29× increase in frame rate and significant improvements in spectra fidelity. This work makes reliable field calibrations and measurements of explosions with Fourier-transform infrared hyperspectral imagers more achievable than before.

## 1. Introduction

The trade-space in remote sensing between spatial, spectral, and temporal resolution is well-known. More explicitly, if higher resolution spatial or spectral data is desired while maintaining the full field of view (FOV) and spectral bandwidth, then it will come at a cost to the temporal frequency of the data. In some instances, this does not present an issue because the scene is static or changes very slowly in relation to even the slowest frame rates of hyperspectral imagers (HSIs) operating at full FOV and spectral resolution. Examples include agriculture, geology, and cityscapes [1,2,3,4,5,6,7]. In these cases, the user has more freedom in the configuration of the instrument in use. However, there are rapidly evolving scenarios that force the user to make more trade-space decisions between temporal, spectral, and spatial resolution.

One such example of fast-transient events is an explosion, which initiates and concludes in less than a second. Fourier transform spectrometers (FTSs) have been employed to remotely sense these fast scenes for decades and for good reason [8,9,10,11,12,13]. FTSs (both rapid scan and step scan) have been used to observe other fast-transient events as well, such as chemical discharges, shock tube experiments, and emission plumes [14,15,16,17,18,19,20]. Chemical discharges and pulsed discharge products have been successfully measured using FTSs in step-scan acquisitions at the microsecond time-scale. The step-scan FTS method relies on the event occurring repeatedly: once at each interferogram measurement [21]. For events such as explosions, where repeated measurements of this quantity are not feasible, the time-resolved step-scan FTS method is not practical. Imaging FTS (IFTS) has been used for measuring gaseous effluent from combustion engines [18]. This is relevant to IFTS use for measuring explosions since both explosions and combustion exhaust plumes share a common spectral region of interest (mid-wave infrared (MWIR)) and molecular constituents (CO_2_, CO, CH_4_, etc.). However, the timescales and temperatures of interest remain drastically different. Rodriguez et al. viewed combustion exhaust plumes with an IFTS by measuring the signal from the plume with a wide-area blackbody (BB) in the background, allowing for measurement of plume transmission and self-emission [18]. Measurements of explosion plumes typically target early, fast-changing, hot signatures [8,9,10,11,12,13].

FTSs, often employing a Michelson interferometer, offer some key advantages [22]. These include sampling all frequencies simultaneously (multiplex or Fellgett advantage) and a larger effective aperture (throughput of Jacquinot advantage) [21]. A Michelson interferometer operates by splitting incoming light into two paths. The light is reflected off mirrors in each path and the two separate paths are then rejoined with one another. The light interferes constructively and destructively depending on the difference in the length of the two paths; this is referred to as the optical path difference (OPD). This OPD is controlled by a moving mirror placed in one of the paths. The point where the difference between the two paths is zero is known as the zero path difference (ZPD). At ZPD, the light from the two paths experiences 100% constructive interference. In order to acquire the scene at the specified spectral resolution, the mirrors must sample both ZPD and MOPD at a minimum.

FTSs are limited, however, by the mechanical movements of the mirror, the number of measurement points per interferogram, and the integration time [23,24]. Further, these studies traditionally use single-pixel FTSs, eliminating the spatial information almost entirely. Another limitation of hyperspectral instruments is the amount of data generated and how quickly that data can be read out. What a single-pixel instrument lacks in spatial information, it gains in temporal information by drastically reducing the data readout time and thereby increasing the frame rate to speeds relevant to explosion signatures (10+ Hz). Though single-pixel FTSs have been used extensively in observing explosions, imaging FTSs (IFTSs) are less common. This is due, in part, to the slow frame rates associated with them relative to the lifetime of the fireball and early plume. The next few subsections will address the parameters linked to frame rates in IFTSs, the challenges that arise from altering these parameters, and scene change artifacts (SCAs).

In particular, SCAs become an issue when the scene changes during the acquisition of the interferogram. This is because standard processing techniques assume the scene is static. A static scene allows for a constant offset to be calculated and used. However, when the scene changes during the interferogram acquisition, the offset is no longer constant. When a constant offset is calculated on this data, the difference is carried into the Fourier transform, and the resulting spectra exhibit oscillations overlaid on it.

This paper presents an approach to increasing HSI frame rates while mitigating the challenges that inherently arise for the purpose of capturing relevant spatial, spectral, and temporal measurements of explosions. In Section 1.1, the parameters that determine frame rates are discussed along with their associated benefits and drawbacks. The drawbacks are addressed through post-processing improvements in updates from the instrument manufacturer and a recently developed method for handling SCAs. This work represents the first use of this correction on explosions or scenes collected outside the laboratory setting. Section 2 describes the chosen parameter settings for observing explosions. Section 3 describes the field experiment where explosions were measured using the HSI and settings described in this paper. Finally, in Section 4, the HSI frame rate improvement matrix is presented with incremental improvement factors and a final frame rate increase from 0.4 fps to 11.3 fps while preserving spectral fidelity. Throughout this paper, the term “frame rate” refers to the number of 3-dimensional hyperspectral cubes generated per unit time.

### 1.1. Parameters Linked to Frame Rate

Frame rates are inversely proportional to the time it takes for the instrument to capture a scene, read out the data, and be ready to capture the next scene. This time-per-frame for IFTSs can be thought of using the following:

(1) $$t_{frame}=\left(t_{int}+max\left[t_{mirror},\;t_{ROIC}\right]\right)\times n_{meas}+t_{external\;readout},\;$$

where $t_{frame}$ is the time from the start of one frame acquisition to the start of the next, $t_{int}$ is the integration time for the measurement at each OPD, $t_{mirror}$ is the mirror displacement time between each OPD, $t_{ROIC}$ is the ROIC readout time at each OPD, $n_{meas}$ is the number of measurement points per interferogram, and $t_{external\;readout}$ is the external readout time. In a continuous scan interferometer, like the one in this study, $t_{mirror}$ only becomes a factor when the ROIC readout time is less than the time it takes the mirror to transit the distance from one OPD to the next. This is because $t_{ROIC}$ and $t_{mirror}$ run concurrently. The variables in Equation (1), with the exception of $t_{mirror}$, can be changed by altering the following parameters:

- Quantity of measurement points taken per interferogram: $n_{meas}$;
  – Spectral resolution;
  – Symmetric vs. asymmetric interferogram;
    * If asymmetric, quantity of measurement points on the short side;
- Integration time: $t_{int}$;
- Readout integrated circuit (ROIC) time: $t_{ROIC}$;
  – Quantity of pixels;
- External Readout time: $t_{external\;readout}$;
  – Amount of data per hypercube;
    * Quantity of pixels;
    * Quantity of interferogram measurement points.

Many of these factors are common to all FTSs, single-pixel and imaging. The number of pixels, which directly impacts the data size and readout time, is the distinguishing factor between the two when it comes to frame rates. The following section will outline some of the background with regard to how and why the above-mentioned parameters impact frame rates.

#### 1.1.1. Impact of Spectral Resolution

The spectral resolution selected by the user determines the minimum value for the maximum optical path difference (MOPD) of the interferometer. The nominal spectral resolution, defined as the full-width at half maximum (FWHM) for an unapodized interferogram, is

(2) $$\Delta \tilde{\nu }=\frac{1.2}{4d}=\frac{1.2}{2\times MOPD},$$

where $\Delta \tilde{\nu }$ is the spectral resolution in wavenumber units [cm^−1^] and *d* is the maximum mirror displacement [cm]. The maximum mirror displacement is one half the MOPD [25]. Application of an apodization function, such as the Hamming window, further broadens the instrument line shape [21]. The spectral resolution directly impacts the frame rate because the MOPD determines the number of measurement points since the mirror displacement step size is fixed. The step size, determined by the Nyquist sampling criterion and the upper spectral limit, is given by

(3) $${\tilde{\nu }}_{max}=\frac{1}{2(2\Delta d)},$$

where ${\tilde{\nu }}_{max}$ is the spectral upper limit in wavenumber units and Δd is the mirror displacement step size [cm]. The Nyquist sampling criterion requires at least two interferogram samples per optical cycle to avoid spectral aliasing. It is evident that the number of measurement points in each interferogram is inversely proportional to the spectral resolution.

#### 1.1.2. Impact of Symmetric vs. Asymmetric Interferograms

The standard operating configuration for a Michelson interferometer is to sample the scene from MOPD to -MOPD (or the reverse), essentially sampling the scene twice. This allows for complete capture of the phase and enables better corrections for any misalignment in the system. However, this requires twice as many points to be measured and twice as much movement of the mirror to occur. This is referred to as a symmetric or two-sided interferogram.

The alternative is an asymmetric interferogram where the scene is measured from MOPD to ZPD + **S**. Where **S** is the short-side distance dependent on a user-defined value for the number of points measured beyond ZPD on the short side of the interferogram. Asymmetric interferograms have the distinct advantage of having far fewer points measured for the same spectral resolution as their symmetric counterparts. However, the downside is the decrease in scene fidelity since asymmetry removes the inherent phase corrections afforded to the symmetric interferograms and increases susceptibility to instrument misalignment. However, since the phase is a slowly changing feature of the scene, it can be approximated by fewer measurement points and then applied to the remaining data. This phase correction is known as the Mertz method and is common in many commercial FTS instruments [26,27,28].

Another disadvantage is the increase in noise equivalent spectral radiance (NESR) in accordance with

(4) $$NESR_{asym}=NESR_{sym}\times \sqrt{\frac{N_{sym}}{N_{asym}}},$$

where $N_{sym}$ and $N_{asym}$ are the number of interferogram measurement points for the symmetric and asymmetric collections respectively. This relationship demonstrates that moving from a two-sided interferogram to a one-sided interferogram yields an increase in noise (a.e.e.). This is derived from the relationship between SNR and the amount of time measuring the scene across the whole scan (SNR $\propto t^{-1/2}$). With constant integration time across the interferogram, the time scales directly with the number of measurement points and thus $SNR\propto N_{meas}^{-1/2}$ [22].

#### 1.1.3. Impact of Integration Time

The impact of integration time on the frame rate is straightforward and quite intuitive but deserves mentioning since it is one of the primary drivers of frame rate. Careful selection of the integration time will balance frame rate and the signal level of the target of interest. Brighter scenes will require faster acquisition to avoid sensor saturation and will lend themselves to faster frame rates. However, dimmer scenes will require longer integration times in order to obtain sufficient signal to distinguish the scene from the noise. In a scene where the temperature changes drastically, this balance proves to be even more challenging.

#### 1.1.4. Impact of Readout Time

Finally, readout time has an impact on the frame rate. Data readout depends on the hardware and the size of the hypercube being saved. There are two readout times to consider: ROIC time and external readout time. The data is initially stored on the ROIC at the time of measurement for a single mirror displacement. This data must be read out to the on-board memory before beginning acquisition at the next mirror displacement. Larger FPA windows and a greater quantity of pixels will take longer for the ROIC to transfer the data to the on-board memory. After completing the capture of a scene as a full interferogram scan, the full hypercube must be transferred from on-board memory to external memory. Larger hypercubes will require longer times for this data readout. While this is directly related to the number of points measured in the interferogram, it is also tied to the number of pixels being captured. The trade-off between spatial fidelity and frame rates must be balanced to meet the user’s needs.

### 1.2. Scene Change Artifacts

Scenes that change brightness during interferogram acquisition present a challenge. SCAs will appear in the Fourier-transformed spectrum. SCAs appear as oscillations on the baseline spectrum. This results because the interferogram offset is traditionally assumed to be constant. The error between the interferogram and its estimate is then Fourier-transformed and the result appears as oscillations in the transformed spectrum. The brightness change may occur due to heating/cooling of the scene or objects transiting the FOV. When considering an explosion, the scene in each pixel is expected to experience both of these changes. Recently, an improved method of offset correction has been developed to remedy the appearance of SCAs [29,30].

## 2. Hyperspectral Imager Setup

The experiments were conducted using a Telops mid-wave extended (MW-E) HSI (Telops Incorporated, Quebec City, QC, Canada). The instrument specifications are detailed in Table 1. The spectral bandwidth is a function of the detector material and the interferogram sampling distance interval, both of which are fixed. The spectral resolution is user-defined within the values specified in the table. The FPA window size is also user-defined, with maximum values displayed in the table. The single-pixel FOV listed can be altered to the user’s desire using an external lens (i.e., the 0.25× lens used in this study). The communication values listed are fixed and describe the ROIC readout rate and external readout rates as 10/100 MB/s respectively. The HSI has 16 data taps in the ROIC, each having 10 MB/s readout rates. The data is stored in 8-bit format. Thus, the ROIC readout time per interferogram measurement is simply the number of pixels divided by 160 MHz.

### 2.1. Asymmetric Interferogram Selection

Asymmetric or one-sided interferograms capture the scene as described in Section 1.1.2 above. The advantage of asymmetric interferograms for capturing fast-transient events is that they nearly double the frame rate by reducing the number of measurement points to nearly half that of a two-sided interferogram. The reduction is not quite half due to the short side of the mirror scan. For 2 cm^−1^ spectral resolution and the default short-side length of 50 measurement points (OPD=0.0316 mm), the asymmetric interferogram is 50.5% the length of the symmetric interferogram of the same resolution (4790 measurement points vs. 9480 measurement points). For 8 cm^−1^ spectral resolution and the default short-side length, the asymmetric interferogram is 52.1% the length of the symmetric interferogram. The short side of the scan is what determines the extent of the phase information for the Fourier-transformed spectra.

#### 2.1.1. Short-Side Length Selection

The default value for the number of measurement points on the short side of an asymmetric interferogram is 50 for the Telops MWIR HSI. However, this value is user-defined and can be adjusted as needed. It was observed that, with the default asymmetric settings, the spectra exhibited oscillatory artifacts. Notably, this occurred even in measurements of a static BB in a laboratory environment. This behavior was unexpected.

**Figure 1:**
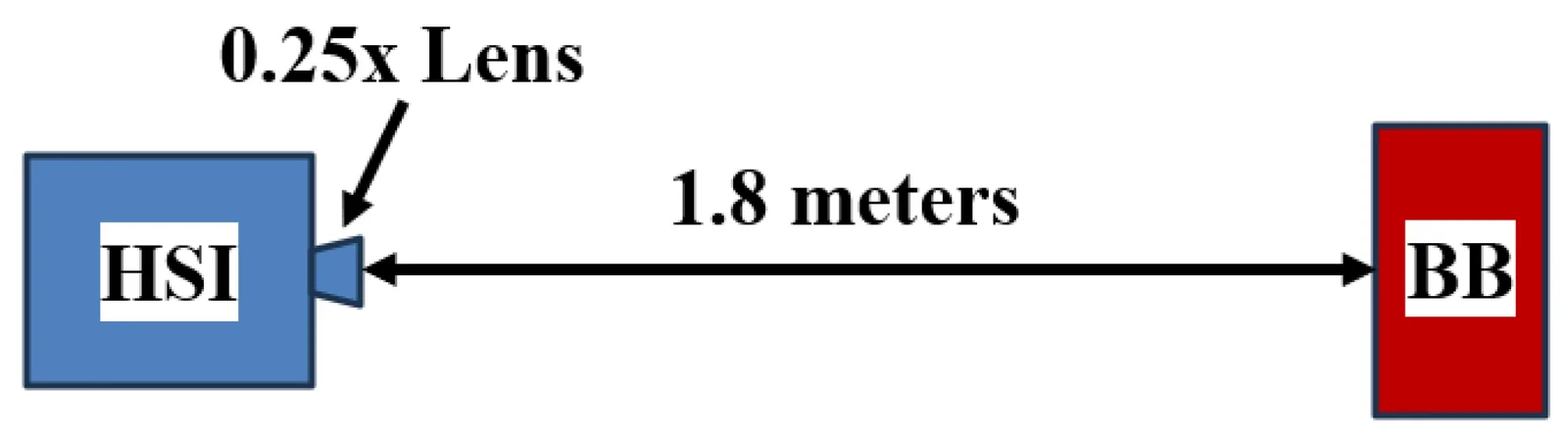
The lab setup for the parametric study of HSI asymmetric interferogram collections.

Since the transformed symmetric interferogram did not exhibit oscillations but the asymmetric one with 50 measurement points on the short side did, it follows that as the short-side length approaches that of a full two-sided interferogram, the transformed spectra will also approach that of the transformed two-sided interferogram. The oscillations in the spectra should approach extinction as the interferogram approaches being fully two-sided. A study was conducted to determine what short-side length was sufficient in eliminating/sufficiently reducing the oscillations. This study was conducted in the laboratory environment with parameters as specified in Table 2 and setup as illustrated in Figure 1. The wide-area BB for this experiment was a 12-inch CI Systems model SR-800N-12HT (CI Systems Ltd., Richardson, TX, USA) [31]. It was set to 200 °C for the measurements. The short-side length was varied to the following values: 25, 50, 100, 200, 500, and 1000 (multiply by $\Delta OPD=6.3295\times 10^{-4}$ mm for OPD). For comparison, the length of one side, from MOPD to ZPD, is 4740 measurement points for a spectral resolution of 2 cm^−1^ on this instrument.

The selection method for optimizing the trade-off between frame rate and short-side length required to reduce oscillations was to determine the knee in the curve of a Concordance Correlation Coefficient (CCC or $\rho_{c}$) plot. The plot visualizes the relationship between $\rho_{c}$ and the number of short-side measurement points beyond ZPD. The coefficient is a measure of how similar two vectors are in both magnitude and shape. A value of one is an exact match. It is calculated using the following equation [32]:

(5) $$\rho_{c}=\frac{2\sigma_{12}}{{\left(\mu_{1}-\mu_{2}\right)}^{2}+\sigma_{1}^{2}+\sigma_{2}^{2}},$$

where $\rho_{c}$ is the correlation coefficient, $\sigma_{12}$ is the covariance of the two vectors, and $\mu_{1}\left(\mu_{2}\right)$ and $\sigma_{1}\left(\sigma_{2}\right)$ are the means and variances of vectors one and two respectively. The comparison of interest is that of the Fourier-transformed asymmetric and symmetric interferograms. The closer the short side of the asymmetric interferogram gets to the length of one side of the symmetric interferogram, the closer $\rho_{c}$ will get to one.

The knee of diminishing returns in the CCC curve was determined using a normalized maximum vertical deviation criterion consistent with the Kneedle knee-detection method [33,34]. This method identifies the knee as the data point with the maximum vertical distance from the endpoint reference line. The method first performs a min-max normalization on the independent and dependent variables, thereby removing the effect of differing axes units. This normalization generates a unit vector reference line (y=x). The distance metric is calculated as

(6) $$D_{i}=\rho_{c,i}^{\prime }-L_{i}^{\prime },$$

where *D* is the distance metric, $\rho_{c}^{\prime }$ is the normalized CCC, and $L^{\prime }$ is the normalized short-side length. The greatest distance indicates the coordinate of maximum deviation from the reference line and marks the transition point between rapid improvements and diminishing returns.

#### 2.1.2. Telops Enhanced Processing

Upon further investigation of the oscillations arising from the use of asymmetric interferograms on static BBs, it was determined that the cause is a variation in the ZPD point in the interferograms across the pixels. However, the processing software assumed a constant ZPD position for all pixels. The incorrect ZPD determination introduces a linear phase error which then translates directly into oscillations/ripples in the transformed spectra. This has been shown in the literature [21,35]. When more short-side measurement points are used, the phase correction becomes more complete and smooths out the oscillatory artifacts even when ZPD is not accurately determined. However, fewer short-side measurement points expose the use of an incorrect ZPD in the calculations. This is why the artifacts were not present in spectra resulting from measurements of fully two-sided interferograms; the phase correction was complete in this case.

The variation in the ZPD point across the pixels may be due to shear or misalignment of the FTS corner cubes. Both of these would cause deviations in the optical path across the FPA. As mentioned, the previous processing software assumed the ZPD to be constant. Thus, the ZPD point was determined by taking the average ZPD point across the FPA and then applying that ZPD offset correction to every pixel. Upon highlighting the issue, Telops updated the software to include a pixel-by-pixel ZPD point determination and phase correction.

### 2.2. Smooth Offset Correction for SCAs

Traditionally, FTIR processing assumes constant brightness and a constant interferogram offset. This assumption is inappropriate for fast-transient targets with large temporal fluctuations in size, temperature, and brightness. Estimating a variable interferogram offset with a constant will lead to error. When the interferogram offset is removed, that error is left as a remainder. It is then Fourier-transformed alongside the interferogram and superimposed on the spectra, giving the appearance of significant noise and/or oscillations. Smooth offset correction (SOC) uses a variable interferogram offset, reducing the error and associated SCAs for targets with fluctuations in brightness and size [29,30]. This correction method was applied to the entire data set.

### 2.3. Reduced Spectral Resolution Selection

Next, the spectral resolution was degraded from 2 cm^−1^ to 8 cm^−1^. This affords a reduction in the number of interferogram measurement points by nearly a factor of four and an increase in the frame rate by nearly the same factor. The spectral resolution determines MOPD in accordance with Equation (2). Thus, lower resolution requires a shorter MOPD which yields fewer measurement points. The ideal improvement would scale linearly; however, the data readout rates and short-side lengths of asymmetric interferograms limit the projected improvement.

The spectral resolution of 8 cm^−1^ was selected in an effort to balance an increase in frame rate as well as maintain sufficient resolution to discriminate the spectral features of molecular constituents such as CO, CO_2_, species of CH, and other potential materials (molecular aluminum). In fast-transient events, the time dependence of the signatures is often one of the strongest determining factors in identification or discrimination [8,10,11,13,36,37,38]. Thus, there is an inherent trade-off between spectral discrimination and temporal discrimination. The balance between the two will depend on the use-case. For instance, determining the time dependence of broadband integrated intensities or temperatures requires much lower resolution (16+ cm^−1^) which would enable frame rates that are faster still [11,13,36,38,39]. However, in cases where molecular constituents play an important role in signature exploitation, resolutions of 8 cm^−1^ or better are often required [40]. That is where this work sits, between the necessity for speed and spectral resolution. It is also worth noting that it has been shown that reduced spectral resolution (smaller MOPD) reduces SCAs, an added but extraneous benefit if SOC is applied [29].

### 2.4. FOV Expander Selection

Employing a 0.25× lens enabled capturing the same full FOV while using 16× fewer pixels. Though this sacrificed spatial resolution of the scene, this trade-off was deemed acceptable in order to achieve increased temporal resolution while keeping the target within the full FOV long enough to capture relevant signatures prior to the brightness falling below the minimum detectable signal. As mentioned previously, the frame rate does not double with halving of the spectral resolution largely because of the bottleneck created by data readout. The size of the window directly impacts the readout time. It is the ROIC readout time that has the largest impact on the total frame rate since it is multiplied by the number of mirror displacement measurement points in the interferogram. The second readout time to consider is the readout from the on-board memory to the external memory in preparation for the next scene. The data for an HSI scene includes header information and the total number of data points. While the data volume of the header can not be altered, the volume of the scene hypercube can be. The total number of data points in the scene is simply N*_x_* × N*_y_* × N*_m_*, where N*_x_* and N*_y_* are the number of pixels in the x-dimension and y-dimensions respectively, while the last dimension is mirror displacement. In studying the time between measurement points as a function of window size, it is clear that the ROIC readout has a large impact on the overall frame rate.

### 2.5. Integration Time

The integration time for the explosions in this study was set to 15 μs based on signal levels and the dynamic range of the FPA for expected temperatures between 450 °C and 1100 °C. These temperatures are based on the expected temperature dynamics of the post-detonation combustion fireball and subsequent cooling plumes that were being observed as part of a larger study. This relatively short integration time enabled faster frame rates well-suited for the rapidly changing scenes.

## 3. Field Experiment Setup

**Figure 2:**
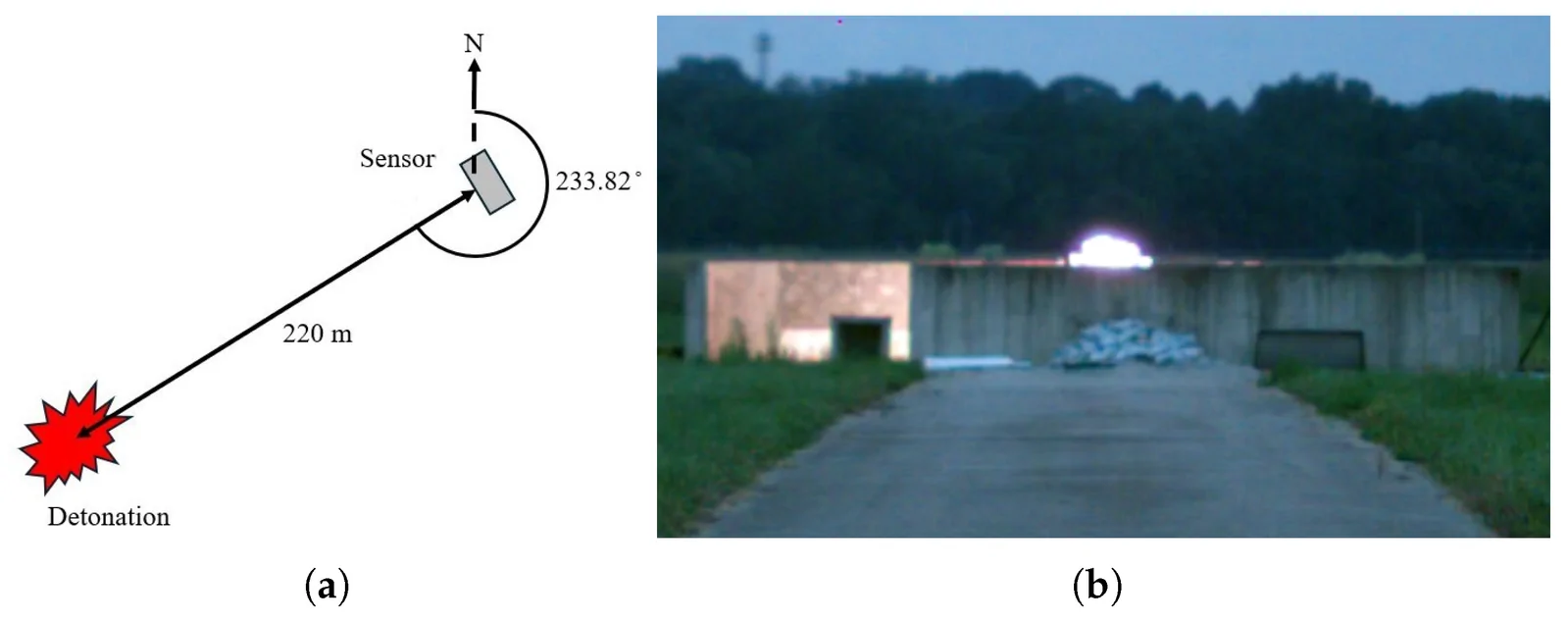
Visual of the field experiment setup: (**a**) a schematic for the field test of explosions. (**b**) Still frame of a C4 explosion captured by a high-speed witness camera during the experiment.

The experiment for which these parameters were tailored included detonations of several high-explosive test articles of various configurations of trinitrotoluene (TNT) and composition-4 (C4). These explosions provided scenes in which the brightness of IFOVs changes rapidly. The rapid changes provide a use case and test bed for the procedures outlined in this paper. Figure 2a illustrates the orientation of the HSI with respect to the detonation location. The standoff distance was assigned by explosive ordnance disposal personnel. The viewing angle was a consequence of the existing facility layout. In addition, several external blackbodies were used for external calibration of the instrument. These include one wide-area BB and two cavity BBs. The wide-area BB was a 6-inch Fluke model 4181 (Fluke Corporation, Everett, WA, USA) set to 450 °C [41]. The cavity BBs were both HGH model RCN1350 N1 (HGH Infrared Systems, Richardson, TX, USA) set to 850 °C and 1100 °C [42]. These temperatures envelop the expected scene temperatures during the first few frames following detonation. These BBs were external to the HSI; therefore, atmospheric corrections were calculated and applied. Path transmission and radiance were determined using a software package called Laser Environmental Effects Definitions and Reference (LEEDR) produced by the Air Force Institute of Technology [43]. Appropriate emissivities for the BBs were accounted for (ε=0.95 for wide-area and ε=0.99 for cavity BBs) [31,41,42].

Safety constraints required the explosives to be set approximately 0.3 m below the top of the containment bunker. This was in order to avoid any potential shrapnel from having a direct line of travel to the observers. This also prevented direct viewing of the detonation early on. The effect of the limitation is visualized in Figure 2b. In practice, this prevents the collection of signatures only in the first 250 μs. This is a very small fraction of the data under consideration and does not prevent analysis. Twenty explosions of TNT and C4 were measured during this experiment; a small sample of the data is included in Section 4.6 for demonstration of the SOC method on SCAs.

## 4. Results

The impacts of tailoring the above-mentioned parameters will vary widely for differing initial conditions and acceptable trade-offs in individual cases. For our application, which involves measuring signatures of high explosives, the greatest increase in frame rates came from the use of the 0.25× lens first. The next greatest improvements came from reducing the spectral resolution and switching to one-sided interferograms. However, the use of one-sided interferograms introduced unforeseen issues in post-processing. These were later corrected. The order in which the changes were implemented and presented is as follows: interferogram symmetry, reduction in FPA window size, spectral resolution, asymmetric short-side length, Telops software update, and SOC for SCAs. The reason short-side length was adjusted last is that it only became a concern after the oscillations appeared in the spectra as mentioned previously. The calculation of the frame rate improvements is also dependent on the order in which the parameter changes were implemented.

### 4.1. Asymmetric Interferograms

The use of one-sided interferograms nearly doubled the frame rate. This was due to the decrease in the number of measurement points and mirror movements required for a single scene by nearly a factor of two. Due to the short-side measurement points, a one-sided interferogram is slightly longer than half of a two-sided one. This also reduced the total data per scene which decreased the data readout time. The experienced increase in frame rate was 1.91× when going from a two-sided to a one-sided interferogram with 2 cm^−1^ spectral resolution.

### 4.2. FOV Expander

The 0.25× lens reduced the data required per mirror displacement measurement by a factor of 16 for the same full FOV (44.8H × 89.6W mrad). This has an outsized impact on the frame rate. For example, the Telops MWIR HSI instrument in this study had a measurement-to-measurement delay of 246 μs between the end of one OPD measurement and the beginning of the next when the window size was 128 × 256 pixels. The same delay for a window size of 32 × 64 was 19 μs. That is an increase in the ROIC readout speed by a factor of 12.9 with a decrease in the spatial resolution by a factor of 4.0. The remaining parameters for this example were as follows: a one-sided interferogram, 50 measurement points on the short side, 2 cm^−1^ spectral resolution in the transformed spectra, and 15 μs integration time. After other factors are brought into consideration, such as the external readout time, the final increase in frame rate for this example is approximately 5.56×, going from 1.340 s/scene to 0.241 s/scene.

Another benefit of windowing the FPA coupled with the use of a 0.25× lens comes from the fact that the individual mirror displacement acquisitions are now closer in time. For fast-transient events, capturing the entire interferogram in a shorter overall amount of time as well as with shorter time between measurements will decrease SCAs and capture a measurement that is more representative of the actual scene.

### 4.3. Reduced Spectral Resolution

Degrading the spectral resolution (2 cm^−1^ to 8 cm^−1^) increased the frame rate approximately 3×. The true improvement is a factor of 1.82× faster when changing the spectral resolution from 2 cm^−1^ to 4 cm^−1^. This corresponded to frame rates of 4.15 Hz and 7.56 Hz respectively. The improvement in frame rate was 1.67× when changing from 4 cm^−1^ to 8 cm^−1^. This corresponded to a frame rate of 12.64 Hz for the 8 cm^−1^ setting. These factors were calculated based on field measurements taken on live, fast-transient scenes in the experiment described in Section 3. The instrument settings were 15 μs integration time, 32 × 64 pixel window size, and one-sided interferograms (default short-side length). The combined improvement in frame rate when moving from 2 cm^−1^ to 8 cm^−1^ is 3.04× for these settings.

### 4.4. Short-Side Length

**Figure 3:**
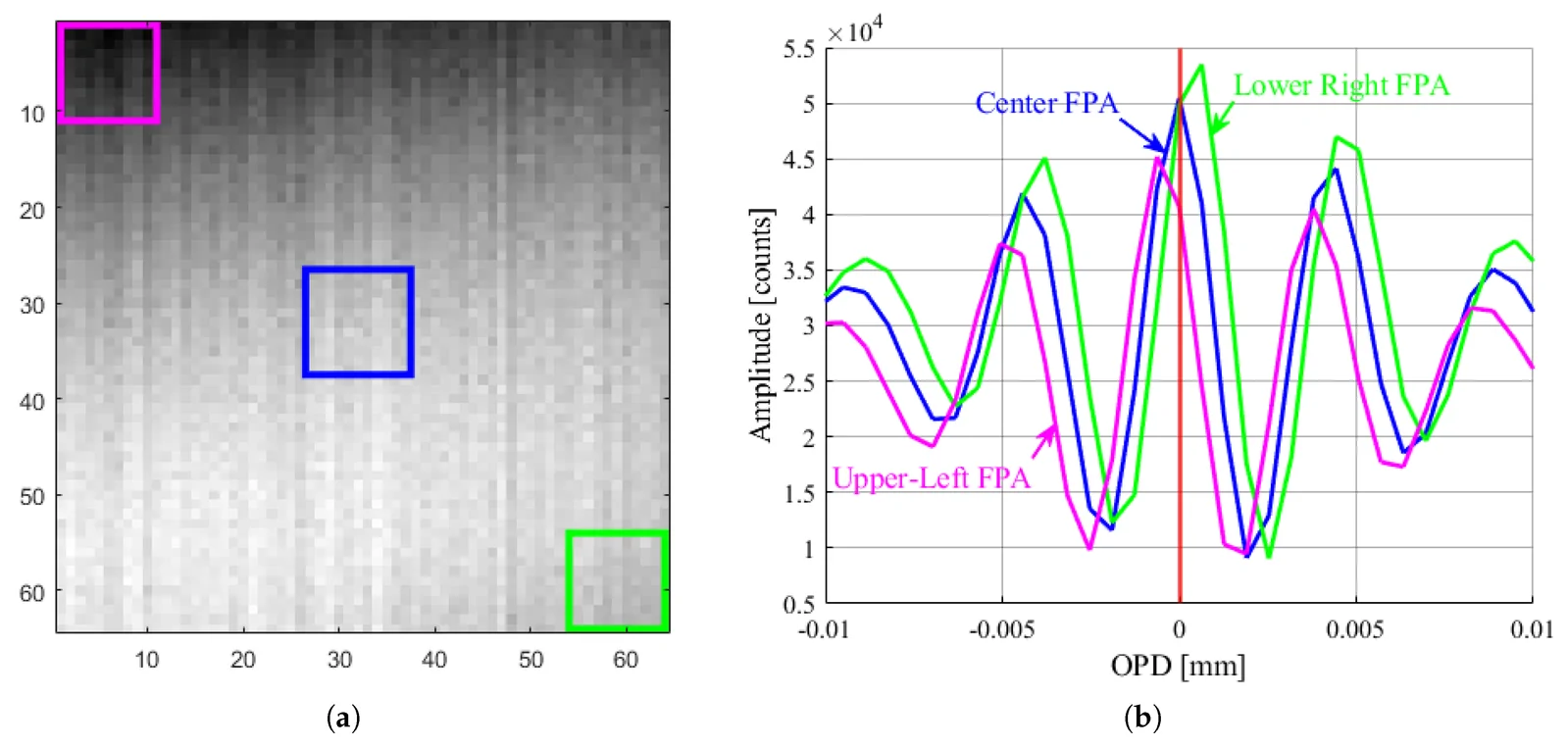
The FPA exhibits a shift in ZPD across the array. (**a**) The shift in ZPD appears to be smooth across the FPA. (**b**) Average interferograms from the 10 × 10 pixel ROIs of corresponding color from the adjacent FPA plot.

**Figure 4:**
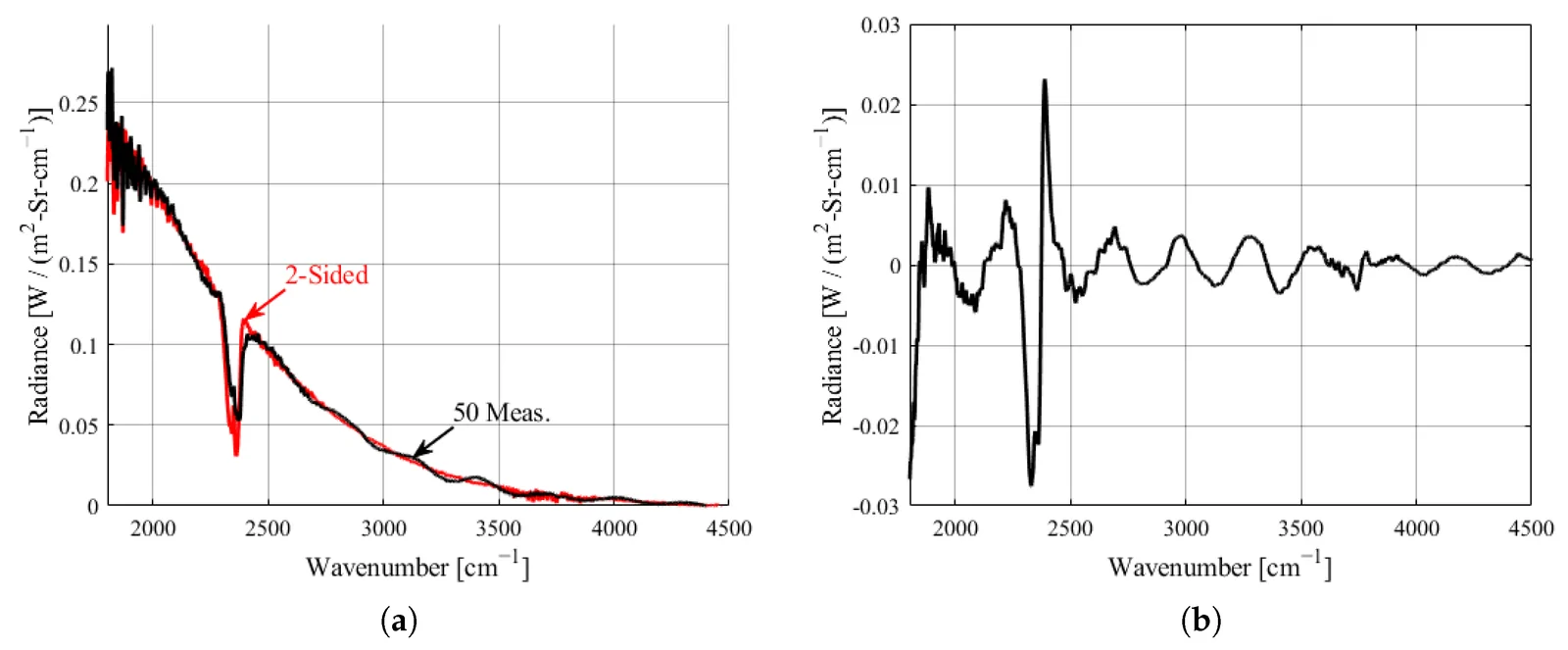
Asymmetric interferograms can introduce oscillations in the spectra if not corrected. (**a**) Comparing the spectra of a 200 °C BB measured using a symmetric interferogram and an asymmetric one with 50 measurement points on the short side of the interferogram. (**b**) The difference between the two spectra in the plot on the left.

Unfortunately, the use of one-sided interferograms introduced increased error in the form of oscillations in the spectra linked to inaccurate knowledge of ZPD across the FPA, as shown in Figure 3 and Figure 4. This illustrates a potential optical alignment problem in the instrument. Figure 3a illustrates the intensity of the interferograms at the assigned ZPD. This corresponds to the red vertical line in Figure 3b. Figure 3b is a plot of the average interferogram for each of the boxes outlined in Figure 3a of corresponding color. This plot shows that the interferograms away from the center of the FPA are being processed with the incorrect ZPD; thus, oscillations can be expected as discussed in Section 2.1.2. The spectra that correspond to the bottom-right corner of the FPA are plotted in Figure 4a alongside spectra taken using a full two-sided interferogram (a.e.e.). The difference was taken and plotted in Figure 4b.

The spectra resulting from the measurement of a full two-sided interferogram lacked any oscillations, as shown by the red plot in Figure 4a. Therefore, it follows that as the length of the short side of an asymmetric interferogram approaches that of a two-sided interferogram, the resulting spectra will also converge. Extending the length of the short side of the interferogram from 50 measurement points (OPD=0.0316 mm) to 200 measurement points (OPD=0.1266 mm) allowed for more robust phase correction of the interferogram while marginally decreasing the frame rate.

**Figure 5:**
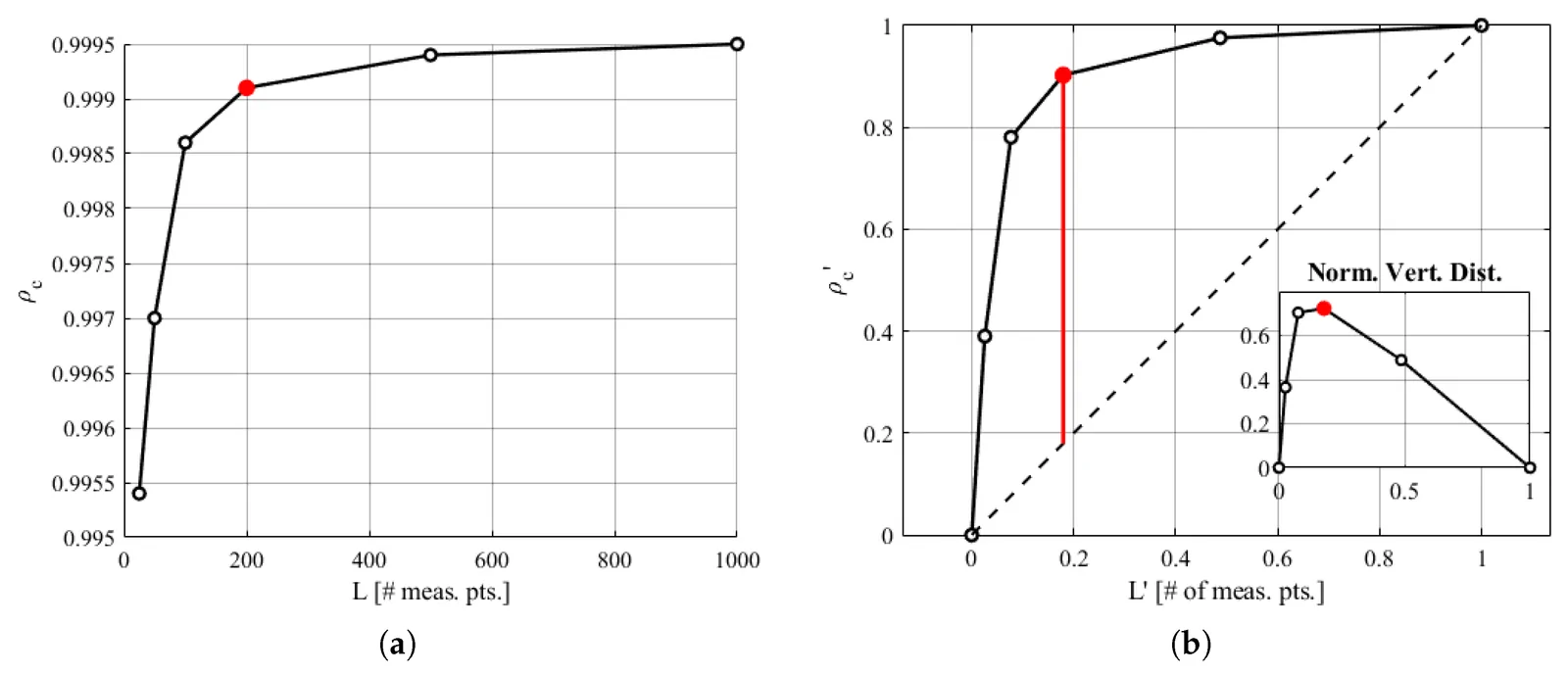
Illustration of how CCC and the Kneedle method were used to select the short-side length of asymmetric interferograms. (**a**) Concordance correlation coefficient as a function of short-side length with selected value highlighted in red. (**b**) Normalized CCC and short-side length with vertical distance as a function of short-side length illustrated in the inset.

While viewing a 200 °C BB, the concordance correlation coefficient was 0.9970 when comparing the transformed spectra of the two-sided interferogram and the default one-sided interferogram (with short-side length of 50 measurement points past ZPD). Increasing the short-side length to 200 measurement points improved the CCC to 0.9991. This value was selected because it was located at the knee of diminishing returns as determined by the normalized maximum vertical distance. This is illustrated in Figure 5 along with the selected knee circle in red. The normalized plot with maximum vertical distance is illustrated in Figure 5b. The corresponding spectra are shown in Figure 5b.

**Figure 6:**
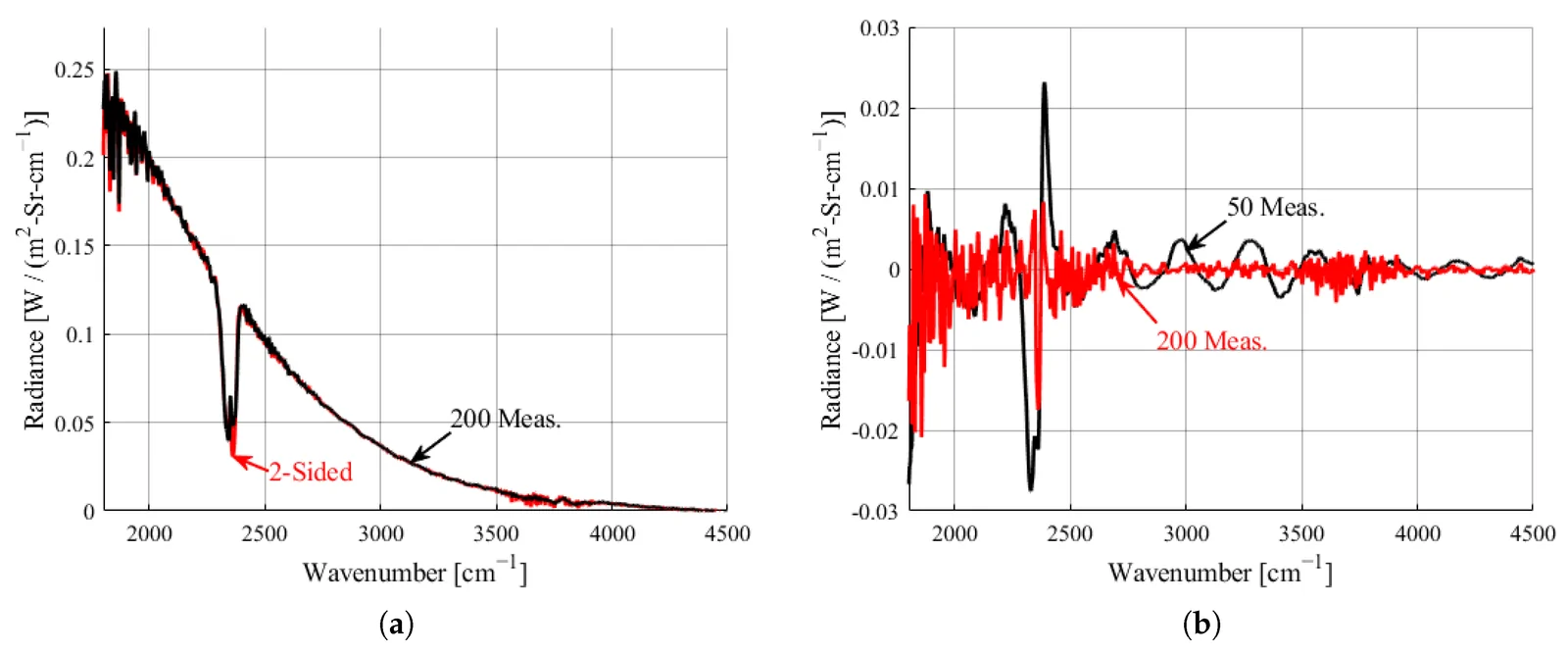
A comparison of the difference between the spectra of a 200 °C BB measured with a two-sided and a one-sided interferogram with 50 and 200 measurement points on the short side. (**a**) Spectra measured by two-sided and one-sided interferograms with a short-side length of 200 measurement points. (**b**) The improvement in the residuals going from 50 to 200 measurement points on the short side of the one-sided interferogram.

For comparison, Figure 6 illustrates improvement in the deviation from the two-sided measurement. The increase in short-side length changed the frame rate by a factor of 0.89 for measurements of 8 cm^−1^ resolution data. While the software update described in Section 2.1.2 and discussed further in the next section largely solved the oscillation issues, understanding how to identify this source of error is vital. This simple fix, in the absence of a more robust solution, such as the Telops pixel-wise correction, also provides utility.

### 4.5. Telops Software Update

**Figure 7:**
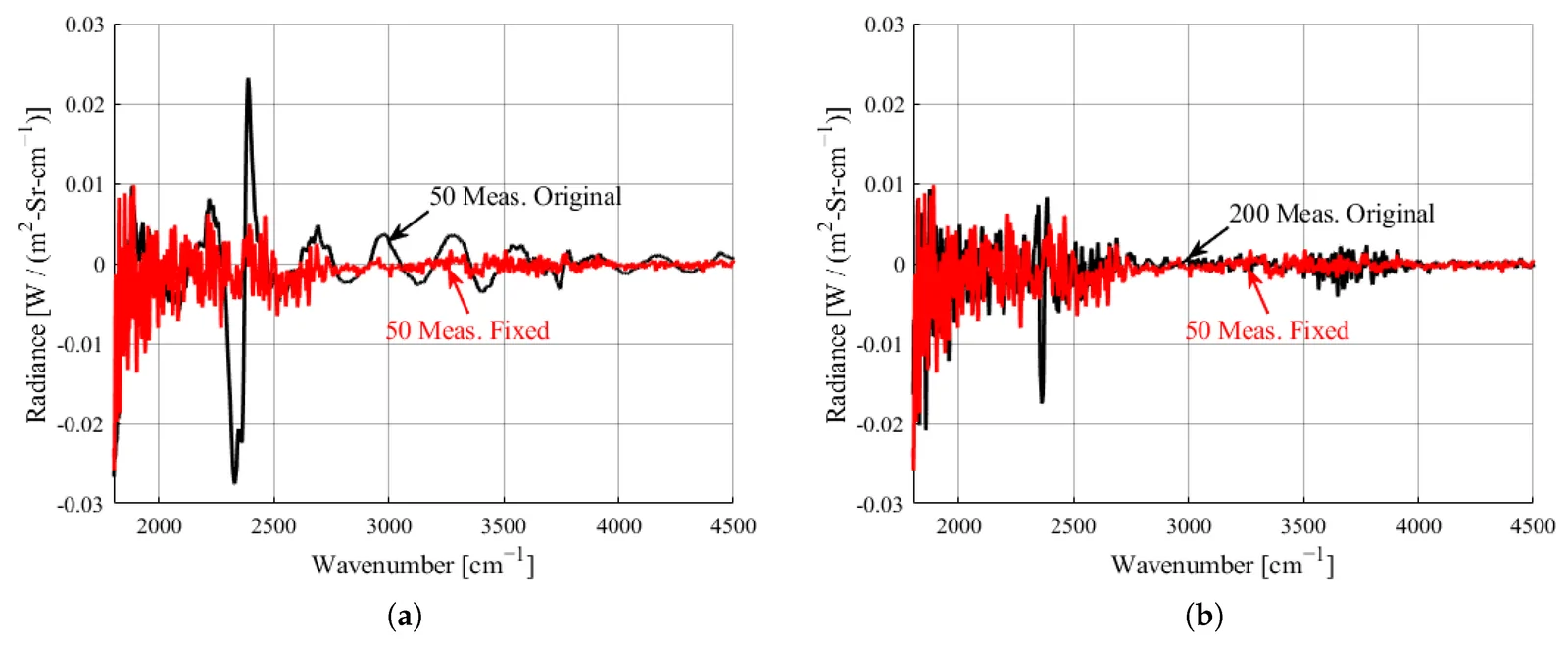
Demonstrating improvements in the deviation from the two-sided measurements. (**a**) Before and after the fix with the default Telops settings. (**b**) Extended short-side length before the fix versus default length after the fix.

Additionally, the Telops Toolbox v5.5.0 software update corrected for the static phase and shear of the instrument on a pixel-wise basis as described in Section 2.1.2. This correction was thorough and yielded results that exceeded the improvements realized through increasing the length of the short side of the interferogram. The Telops fix achieved good accuracy in just 50 measurement points past ZPD (the default value). The results are visualized in Figure 7.

**Figure 8:**
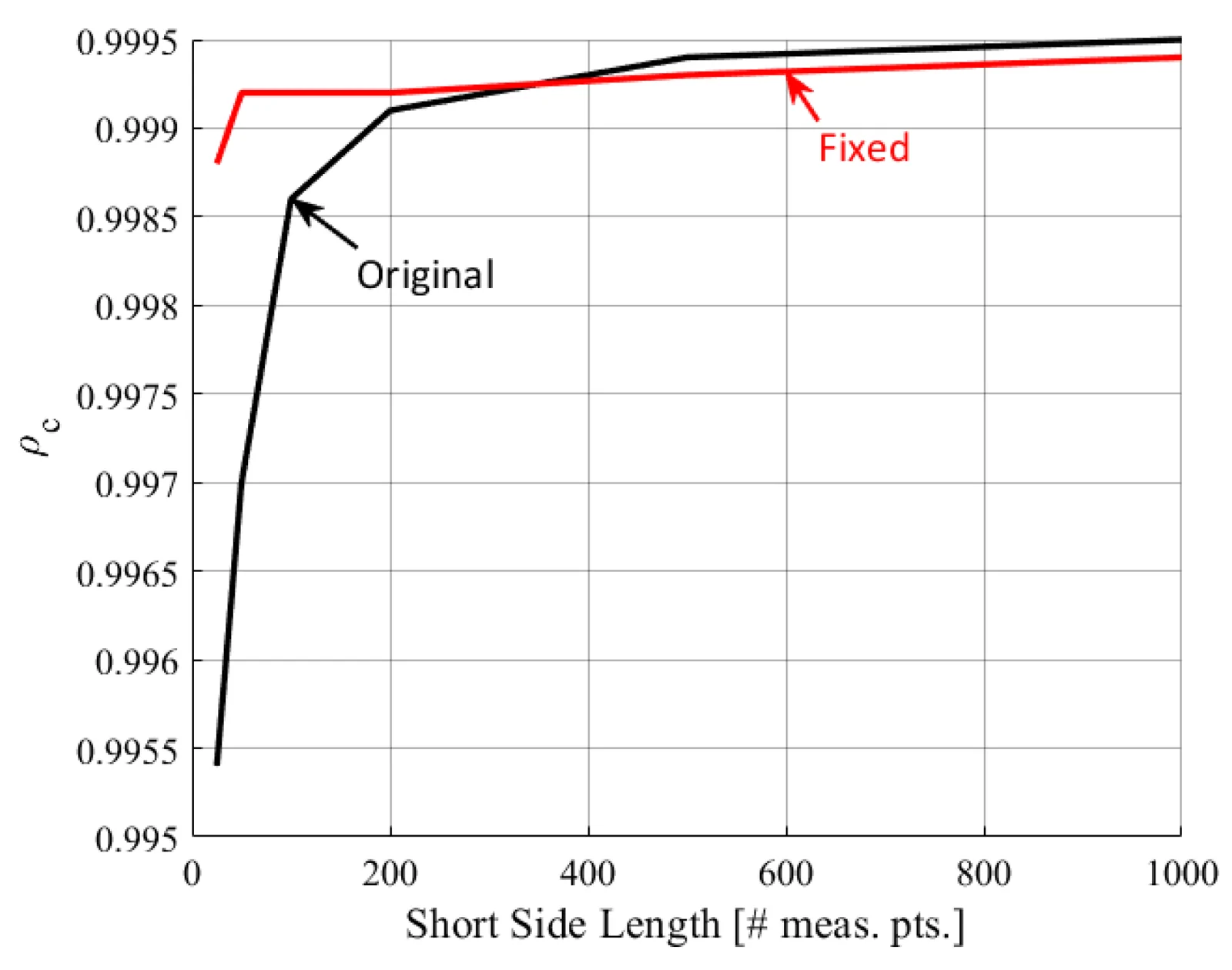
Concordance correlation coefficient as a function of short-side length for both the original processing and the updated Telops software.

When compared to the original CCC values, there is an advantage to using both longer than default short-side lengths and the updated Telops software (see Figure 8). However, the advantage of extended short-side length is dramatically reduced with the updated Telops processing. With the fix, spectra with one-sided interferograms taken with the default settings (50 measurement points on the short side) yield $\rho_{c}=0.9992$ when compared to those taken with two-sided interferograms.

Where this software update is available, it is safe to resume use of the default short-side length in one-sided interferograms. In instances where this is not available or the ZPD offset correction is unknown, extending the short-side length is advised.

### 4.6. SCAs and the SOC

**Figure 9:**
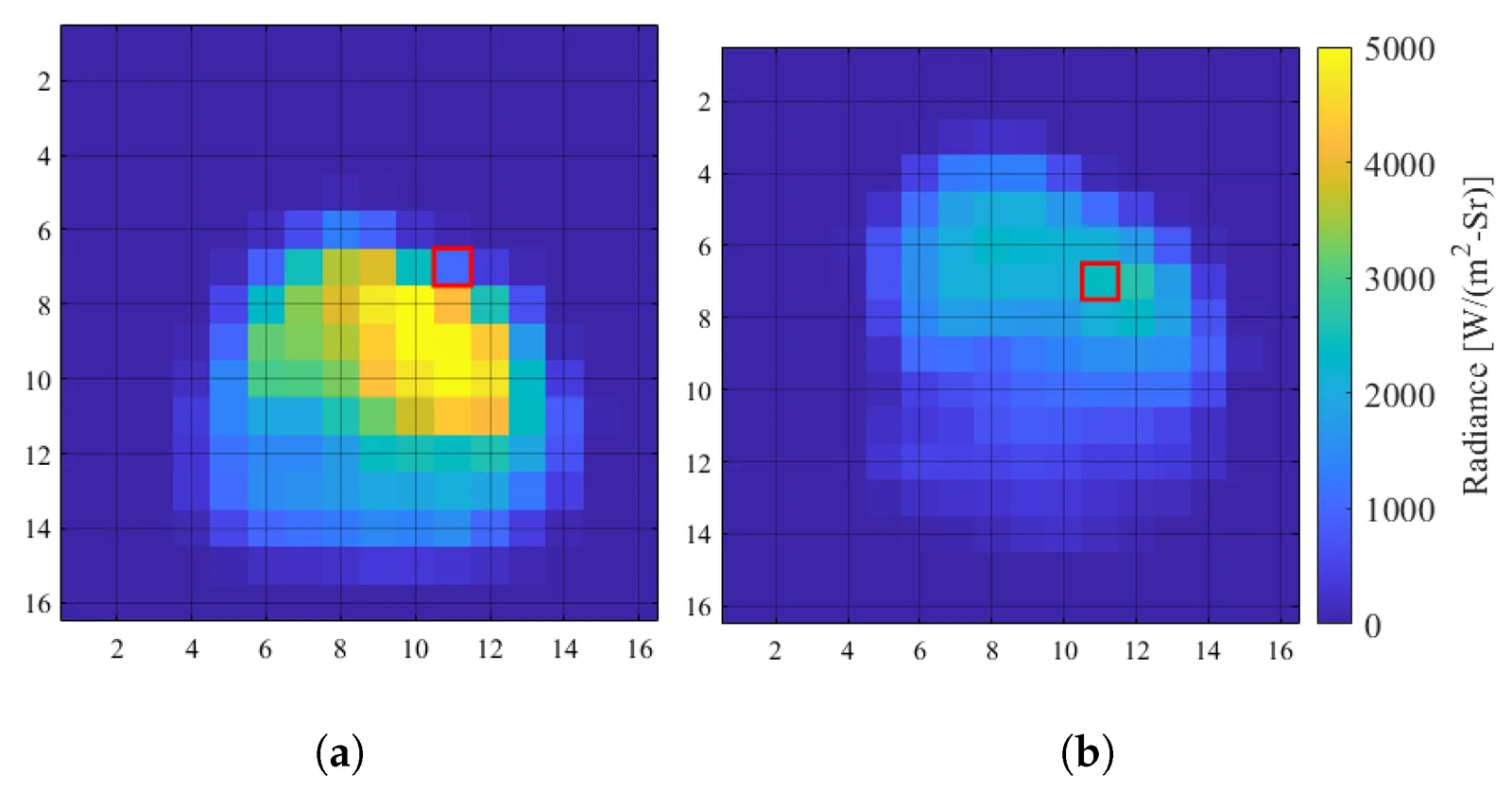
The integrated radiance image for a post-combustion plume of a high-explosive detonation as it cools and moves through the FOV with a pixel of interest highlighted with a red border. (**a**) The radiance image associated with the plots in Figure 10 taken at time $t_{1}$ and (**b**) the radiance image associated with time $t_{1}+0.089$ s.

Another important factor in processing fast-transient scenes is that they are susceptible to SCAs. However, the SOC developed by Wilson dramatically reduces these oscillatory artifacts in the spectra. Fortuitously, the previously applied reduction in spectral resolution was also demonstrated as an effective means for reducing SCAs, though not to the same extent as the SOC [29]. To demonstrate the improvements from the SOC method, the pixel indicated by the red square in Figure 9 has been selected. In this instance, the explosion was a one-pound charge of C4 doped with aluminum shavings. The plume is traveling up and to the right in this figure.

**Figure 10:**
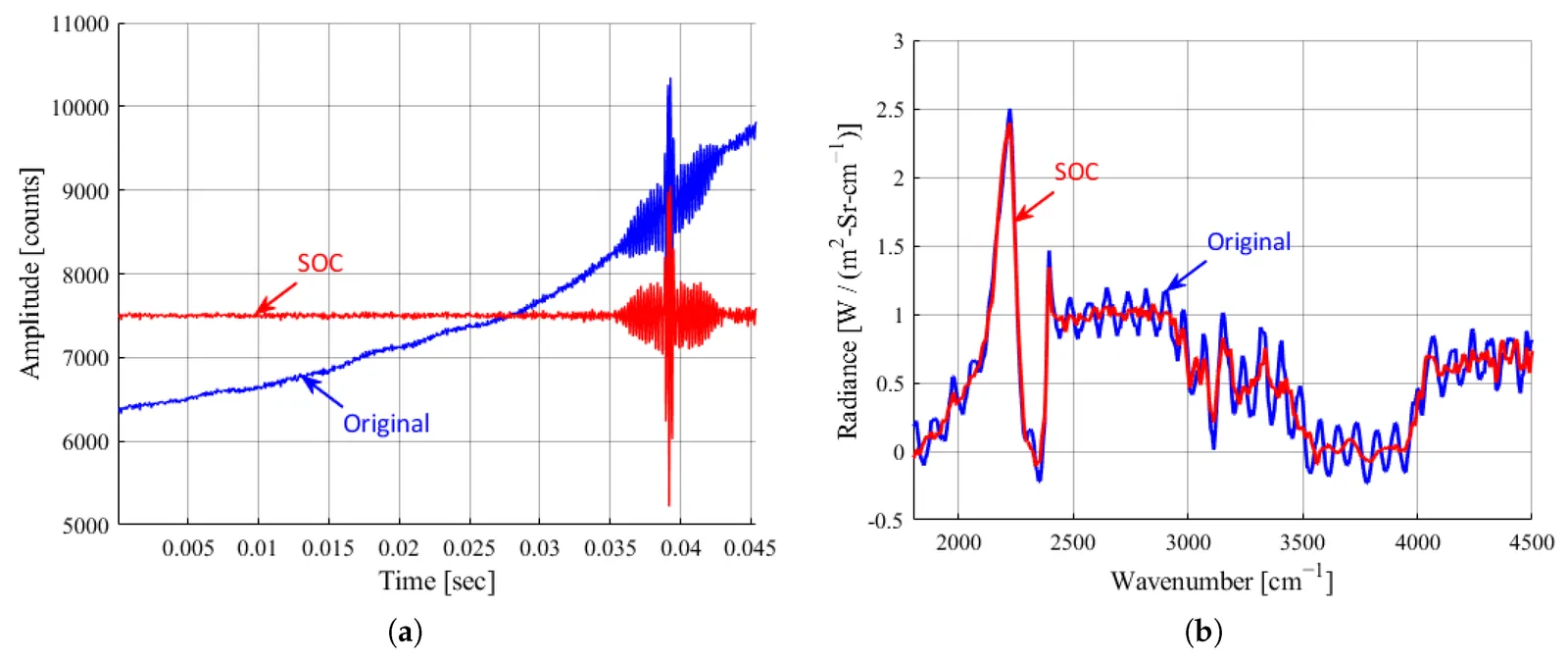
Demonstrating removal of SCAs using SOC on explosion data. (**a**) The interferogram from a select pixel before and after application of SOC. (**b**) The corresponding spectra before and after application of SOC.

The interferograms and spectra in Figure 10 were taken from the previously indicated pixel in Figure 9. The measured interferogram ramps up, signifying an increase in the baseline signal strength despite the fact that the mass is cooling. The hot mass is entering the IFOV, increasing the measured signal despite the fact that the mass itself is cooling. The SOC flat-lines the interferogram and the resulting spectra experience a dramatic improvement with the removal of the drastic oscillations that were present for the uncorrected data.

**Figure 11:**
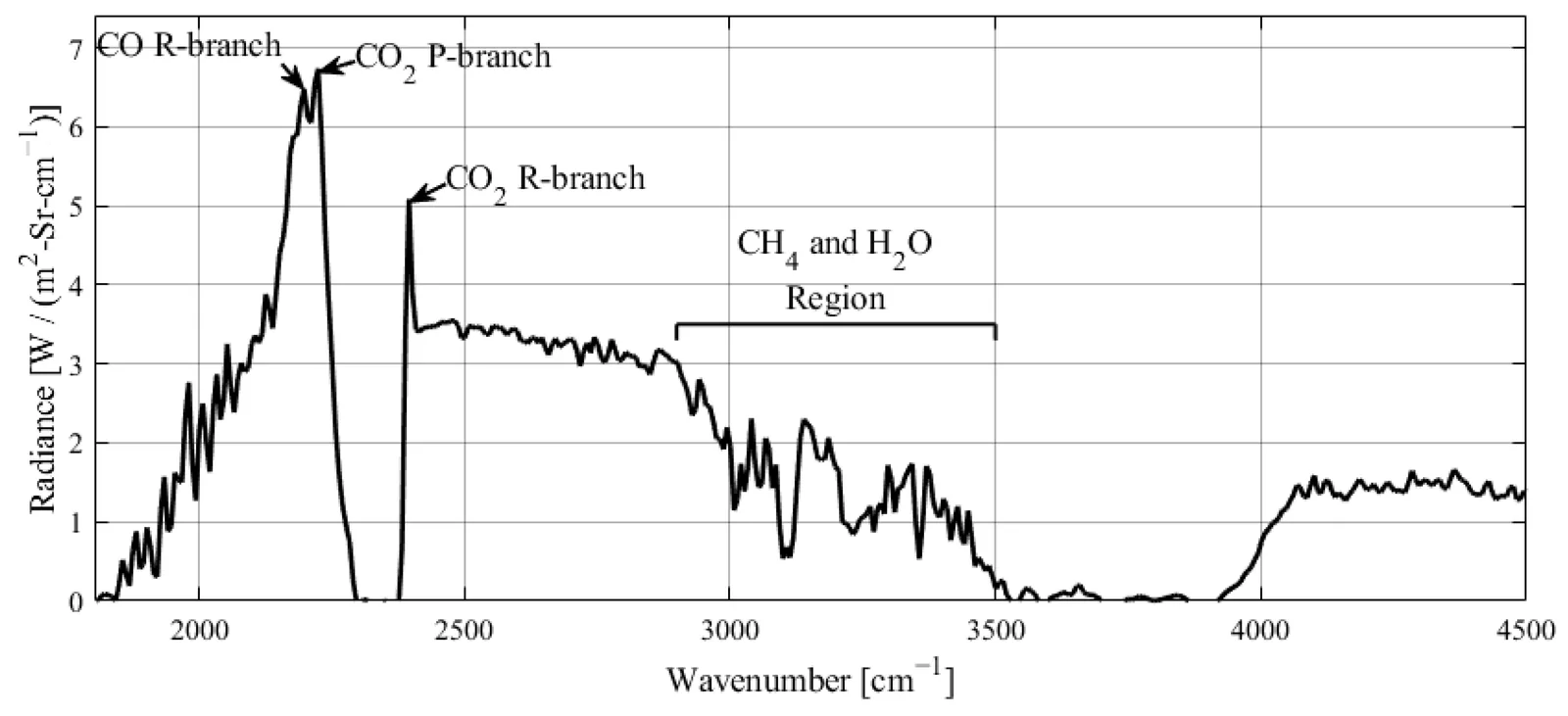
A single-pixel spectrum of a C4 explosion captured and processed under the parameters outlined in this paper.

Previous work provides further insight as well. The extracted SOC gives a high-temporal-fidelity broadband brightness measurement which may prove useful in other diagnostics. Additionally, Wilson has shown the Fourier-transform of the interferogram is the spectrum of the scene at the time of ZPD [29]. It is reasonable to conclude this remains true for measurements of explosions since the measured spectra in this study are largely consistent with those in other explosion studies [8,9,13,37,39]. These explosion spectra are characterized by an overall broadband emission in the MWIR with selective emissions of hot CO and CO_2_ (byproducts of combustion) evidenced in the sharp features surrounding the CO_2_ $\nu_{3}$ band centered at 2349 cm^−1^ and fundamental CO band R-branch at 2170 cm^−1^. The presence of CH and H_2_O emissions, as shown in Figure 11, along with CO and CO_2_, is exactly what is expected from combustion of hydrocarbon fuels such as TNT and C4 [44,45,46]. This spectrum is measured from the scene approximately 0.125 s after detonation and produces a two-color temperature of 655 °C based on the spectral radiances at 2500 cm^−1^ and 2800 cm^−1^. This temperature falls within a reasonable range for this time-frame and this explosion (one pound of C4 with machined aluminum shavings).

### 4.7. Combined Results

Altogether, the combined measures implemented in this method improved the HSI frame rate by a factor of 29 while also eliminating oscillatory artifacts. The intermediate and combined improvements can be reviewed in Table 3. The initial configuration of the instrument, associated with a 0.390 Hz frame rate, is:

- Interferogram symmetry: fully two-sided (symmetric);
- Window size: 128 × 256 pixels;
- IFOV: 0.35 mrad;
- FOV: 44.8 × 89.6 mrad;
- Spectral resolution: 2 cm^−1^;
- Integration time: 15 μs.

The final configuration of the instrument, associated with a 11.31 Hz frame rate, is:

- Interferogram symmetry: one-sided (asymmetric);
- Short-side length: 200 measurement points;
- Window size: 32 × 64 pixels;
- IFOV: 1.40 mrad;
- FOV: 44.8 × 89.6 mrad;
- Spectral resolution: 8 cm^−1^;
- Integration time: 15 μs.

## 5. Discussion

Historically, HSI instruments have not been considered for use in capturing explosions due to their relatively slow frame rates while operating in traditional configurations. Frame rates can be increased through reduction in scene data sizes and shortening measurement times. These can be accomplished through the following:

- Using one-sided interferograms;
- Reducing spectral resolution;
- Reducing the number of pixels.

However, operating with one-sided interferograms increases susceptibility to artifacts in the transformed spectra linked to imprecise knowledge of the ZPD. Through increased short-side length and use of a Telops software package update (Telops Reveal Toolbox v5.5.0) which applies a pixel-wise ZPD offset correction, these artifacts were largely mitigated. This work also identified errors in processing software for a suite of commercially available instruments which resulted in updates to that software that resolve these problems. Unfortunately, SCAs persist due to the rapid change in these explosive scenes. However, the SOC dramatically reduces SCAs even for highly dynamic scenes like these. This work represents the first use of the SOC in scenes collected outside the laboratory setting, demonstrating its utility for explosions and other fast-transient events.

While the parameter settings presented in this work were tailored to a specific experiment. The principles explored can be generalized to measuring explosions of various configurations. For example, larger explosions will maintain brighter signatures for longer. As such, a shorter integration time can be used and capture more hypercubes before the signal drops below minimum detectable limits, both because this will increase frame rates and because the detectable signal persists for longer. Regardless of the explosion, reducing the number of pixels will greatly increase the frame rate. However, the FOV must contain the explosion signature through the decay below detectable limits in order to maximize utility. Thus, the size of the explosion, distance to target, lenses, and FOV window options must be balanced to improve data collection in this parameter space for particular applications. The use of asymmetric interferograms, pixel-wise ZPD offset corrections, and SCA SOC will prove extremely helpful in all cases of remote sensing of explosions and other fast-transient events with IFTSs.

Altogether, these configuration and post-processing techniques have enabled a frame rate of 11.3 fps, a nearly 30× increase in the temporal fidelity of the Telops MWIR HSI instrument, while maintaining 8 cm^−1^ resolution, 15 μsec integration time, and a window size of 32 × 64 pixels. This broadens the application space of HSI instruments into a realm previously dismissed due to the inherent challenges for which a satisfactory and more comprehensive solution has not been presented.

## Figures and Tables

**Table 1:** Instrument specifications for the Telops MW-E HSI used in this work.

Parameter	Value(s)
Spectral bandwidth	1800–6667 cm^−1^
Spectral resolution	0.25–150 cm^−1^
Step size (ΔOPD)	$6.3295\times 10^{-4}$ mm
FPA format	320 × 256 pixels
Single-pixel FOV	0.35 mrad (with 1× lens)
Communication	10/100 MB/s

**Table 2:** Experiment parameters for determining short-side dependence of oscillations in the spectra.

Parameter	Value(s)
BB temperature	200 °C
Spectral resolution	2.00 cm^−1^
FPA format	64 × 64 pixels
Integration time	20 μs
Lens	0.25×
IFOV (with 0.25× lens)	1.40 mrad

**Table 3:** HSI parameter changes and their impact on frame rate.

Parameter	Parameter Change	Frame Rate Before	Frame Rate After	Improvement Factor
Symmetry	Symmetric to asymmetric	0.390 Hz	0.747 Hz	1.915
Window size	128 × 256 to 32 × 64	0.747 Hz	4.152 Hz	5.558
Spectral resolution	2 cm^−1^ to 4 cm^−1^	4.152 Hz	7.557 Hz	1.820
	4 cm^−1^ to 8 cm^−1^	7.557 Hz	12.64 Hz	1.673
Short-side length	50 meas. to 200 meas.	12.64 Hz	11.31 Hz	0.895
Combined				29.01

## Data Availability

The datasets presented in this article are not readily available because they are not cleared for public release. Requests to access the datasets should be directed to James Stofel.

## Abbreviations

The following abbreviations are used in this manuscript:

a.e.e.	All else equal
BB	Blackbody
C4	Composition-4
CCC	Concordance correlation coefficient
FOV	Field of view
FPA	Focal plane array
FTIR	Fourier-transform infrared
FTS	Fourier-transform spectroscopy
FWHM	Full-width half-maximum
HCAM	Hyperspectral camera
HSI	Hyperspectral imager
IFOV	Instantaneous Field of View
IFTS	Imaging Fourier-transform spectroscopy
LEEDR	Laser environmental effects definition and reference
MOPD	Maximum optical path difference
MWIR	Mid-wave infrared
NESR	Noise equivalent spectral radiance
OPD	Optical path difference
ROIC	Readout integrated circuit
SCA	Scene-change artifact
SNR	Signal-to-noise ratio
SOC	Smooth offset correction
TNT	Trinitrotoluene
ZPD	Zero path difference
